# Physical Seed Dormancy in Legumes: Molecular Advances and Perspectives

**DOI:** 10.3390/plants13111473

**Published:** 2024-05-27

**Authors:** Zhaozhu Wen, Xuran Lu, Jiangqi Wen, Zengyu Wang, Maofeng Chai

**Affiliations:** 1 Key Laboratory of National Forestry and Grassland Administration on Grassland Resources and Ecology in the Yellow River Delta, College of Grassland Science, Qingdao Agricultural University, Qingdao 266109, China; 2 Qingdao Key Laboratory of Specialty Plant Germplasm Innovation and Utilization in Saline Soils of Coastal Beach, College of Grassland Science, Qingdao Agricultural University, Qingdao 266109, China; 3 College of Agronomy, Hunan Agricultural University, Changsha 410128, China; 4 Institute for Agricultural Biosciences, Oklahoma State University, Ardmore, OK 73401, USA

**Keywords:** domestication, dormancy, hard seededness, legumes, seed coat, water permeability

## Abstract

Physical dormancy of seeds is a form of dormancy due to the presence of an impermeable seed coat layer, and it represents a feature for plants to adapt to environmental changes over an extended period of phylogenetic evolution. However, in agricultural practice, physical dormancy is problematic. because it prevents timely and uniform seed germination. Therefore, physical dormancy is an important agronomical trait to target in breeding and domestication, especially for many leguminous crops. Compared to the well-characterized physiological dormancy, research progress on physical dormancy at the molecular level has been limited until recent years, due to the lack of suitable research materials. This review focuses on the structure of seed coat, factors affecting physical dormancy, genes controlling physical dormancy, and plants suitable for studying physical dormancy at the molecular level. Our goal is to provide a plethora of information for further molecular research on physical dormancy.

## 1. Introduction

The development of agriculture is a key point in human history, and one of the core elements in agriculture is the evolution of new forms of plants and the domestication of crops [1,2]. In the process of plant evolution and adaptation, seed dormancy has played an important role, as it determines the beginning of the new generation [3,4,5]. In the long history of evolution, in order to maintain their survival capacity, plant seeds have evolved various forms of dormancy to adapt to the complex and changing climate and environments [6,7]. Despite its merits in plant evolution, seed dormancy is not a favorable feature in agricultural production, mainly due to its effects on seed germination. Seed dormancy is primarily classified as physiological dormancy (PD), morphological dormancy (MD), morphophysiological dormancy (MPD), physical dormancy (PY), and physical plus physiological dormancy (PD + PY) [8,9]. Though physical dormancy has been reviewed many times over the years [10,11], compared to the well-studied hormone-mediated physiological dormancy in Arabidopsis thaliana or cereals, only a few articles have been published on physical dormancy regarding molecular aspects [8,11,12,13].

Physical dormancy is caused by the impermeability of the seed coat (or fruit coat), which prevents seed germination [3,13,14,15]. Physical dormancy exists in at least 18 angiosperm families including most legume crops [3,9,16]. To date, research on physical dormancy has primarily focused on leguminous plants, such as soybean, Medicago truncatula, pea (*Pisum sativum*), and other legume crops [3]. This article systematically reviews the literature on physical dormancy, summarizing the structure of seed coat, the seed coat compositions influencing physical dormancy, the genes controlling physical dormancy, and the plants suitable for studying physical dormancy at the molecular level.

## 2. Seed Coat Organ for Physical Dormancy

In species exhibiting physical dormancy, a significant structure known as the hard seed coat covers the seed to prevent water from entering. Research on physical dormancy has primarily focused on the morphological structure and composition of the seed coat, as well as methods for breaking the impermeable seed coat and cuticle [17,18].

### 2.1. Morphological Characteristics of Seed Coat on Physical Dormancy

Morphological observations indicate that seed hardness is related to the structure of the palisade and epidermal layers [19]. The epidermal cells of the seed coat are smooth, with a regular dome-like arrangement [20]. A number of studies suggest that physically dormant seeds have specific morphological characteristics, with the impermeable seed coat primarily consisting of a hardened layer of intact cells, known as the palisade layer [20]. When comparing the non-hard and hard seeds of lupin beans, it has been found that hard seeds have a thicker palisade layer [21].

The palisade layer in the seed coat of *Trifolium repens* and alfalfa is covered by a cuticle layer, which increases the seed hardness [22]. The impermeable cuticle layer of a hard-seeded seed coat is essential for physical dormancy. Scratching the seed coat destroys the cuticle layer, making the impermeable seed permeable [23]. The impermeability of hard soybean seeds can also be attributed to the intact cuticle around the seeds [24,25].

The palisade layer is composed of sclerenchyma cells with thick secondary walls. The most common type of sclerenchyma cells are macrosclereids or Malpighian cells [26]. When the palisade layer of the *M. truncatula* seed coat is observed under a microscope, a bright line crossing the cells is clearly visible, which is known as the light line [27,28]. The light line was identified as the major barrier to water penetration in dormant pea seeds [29]. Cells underneath the palisade layer are hourglass cells. Analysis of the seed coat sections of *Trifolium pratense* showed that the non-hard-seeded seed coat tends to have short and round-shaped hourglass cells, whereas the hard-seeded seed coat has long hourglass cells [30]. Parenchyma cells are located at the bottom layer of the seed coat, adjacent to the endosperm, and are transient nutrient storage organs [31,32,33].

**Figure 1:**
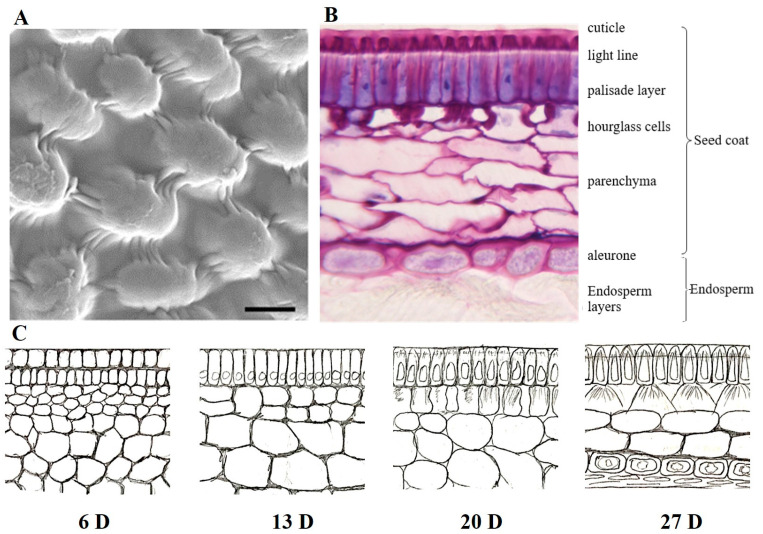
Seed coat of *M. truncatula.* (**A**). Scanning electron micrograph of epidermal cells, scale bar = 10 μm. (**B**). Cross-sections of the seed coat at the stage of maturation before desiccation. (**C**). Cartoons depicting cross-sections of seed coat development in different development stages: embryogenesis (6D), seed filling (13D, 20D), maturation drying (27D).

The primary path of water entry into the seed with physical dormancy includes strophiole, hilum, micropyle, and small dispersed fissures [24,29]. During the entire seed development process of *M. truncatula*, the palisade layer of the outer envelope elongates radially, and the cell wall thickens, ultimately leading to the formation of tightly packed giant hard-shell layers, which increase seed hardness (Figure 1) [34]. The thickness and arrangement of these layers can vary among species and affect seed hardness. Seeds with a thicker palisade layer and intact epidermis tend to have higher levels of physical dormancy.

### 2.2. Composition of Seed Coat and Physical Dormancy

The impermeability of the seed coat cannot be explained solely by its thickness [35]. In addition to anatomical differences in the seed coat, the chemical composition of the seed coat also varies. The palisade layer, which is composed of macrosclereid cells with thick secondary walls, is impermeable to water because of the presence of hydrophobic substances such as cutin, lignin, quinones, pectins, suberin, and wax [21,36,37]. Owing to genetic and environmental factors, the composition of seed coats differs in different species [38].

#### 2.2.1. Lipids

**Figure 2:**
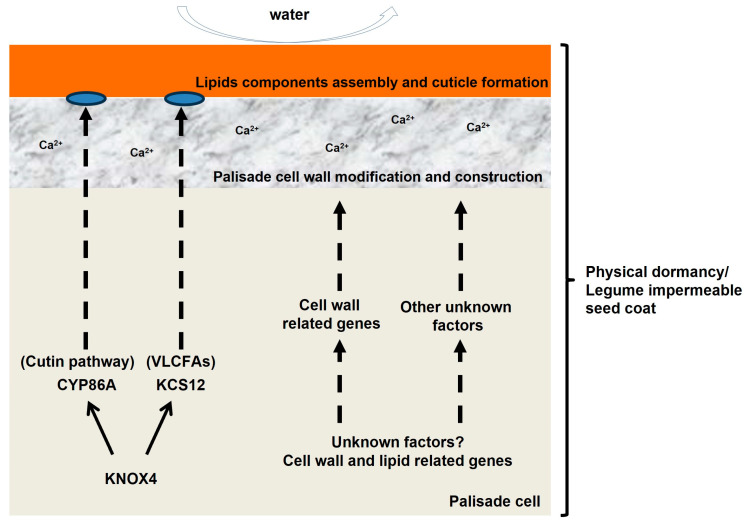
A proposed model for seed coat development in *M. truncatula.* The schematic model depicts known and potential genes and other factors involved in physical seed dormancy in legumes.

In legumes, such as alfalfa, seed permeability is regulated by changes in extracellular lipid biosynthesis [34]. Chemical composition analysis has shown that impermeable soybean seeds contain more hydroxylated fatty acids than permeable seeds [25]. Chemical analysis identified a seed coat-specific cutin with unusual chemical composition that lacks typical mid-chain hydroxylated fatty acids but is relatively rich in other types of hydroxylated fatty acids [25]. The cuticle of an impermeable soybean cultivar contains a disproportionately high amount of hydroxylated fatty acids compared with that of permeable ones. According to Shao et al. [25] and Ma et al. [24], the difference between hard and soft soybean seeds is based on the composition and continuity of the outermost seed cuticle and the presence/absence of small cracks in cuticles. A deficiency in hydroxylated fatty acids may alter the cuticle layer permeability and is a possible explanation for the change in physical dormancy in *M. truncatula* [20]. A significant decrease in the content of very-long-chain fatty acids (VLCFAs) affects the formation of seed physical dormancy in *M. truncatula* (Figure 2) [39]. Electronically driven micromanipulation (EDM) and (matrix-assisted) laser desorption/ionization mass spectrometric ((MA)LDI-MS) analyses revealed that the long-chain hydroxylated fatty acid (HLFA) content in the seed coat of the dormant (wild) pea genotype (JI64) was significantly higher than in their counterparts treated with micropeeling [40]. These results confirm the accumulation of HLFA in the outermost layers (cutin).

#### 2.2.2. Polyphenolics

Many genes related to polyphenol biosynthesis are expressed or even specifically expressed in the seed coat [34]. These genes include MYB–bHLH–WDR (MBW) transcription factor complex as well as some structural genes. Polyphenols such as procyanidins and anthocyanins also accumulate in the seed coat, especially in leguminous plants. Therefore, their impact on the development of the seed coat has attracted attention.

The outer layer cells of the pea seed coat accumulate a large number of polyphenolic compounds that can affect seed permeability when oxidized [5,10]. In pea seeds, the excessive accumulation of gallocatechin in the hilum also alters the permeability of the seed coat [41]. Normalized intensities of signals (NS) of particular polyphenols, namely (epi) gallocatechin and luteolin, are significantly higher in the seed coat of non-dormant (domesticated) genotypes than that in dormant (wild) genotypes [40]. When comparing non-dormant and dormant recombinant inbred lines in chickpea, significant differences were observed in the contents of phenolic acids and flavonoids [42].

#### 2.2.3. Other Components

Physical dormancy is featured with tightly packed epidermal palisade cells. Irreversible cell wall loosening is an important step during seed germination followed by water uptake [43]. Plant cell wall loosening and weakening of the seed coat are affected by various factors and substances [44]. The maintenance of cell wall integrity is vital for the formation of physical dormancy. Increased deposition of β-1,3-glucans (callose) and β-1,4-glucans in the seed coat during seed maturation is associated with increased physical dormancy [45,46].

Ca^2+^ is an important component of the cell wall and plays a significant role in physiological plant regulation, stress tolerance, signal transduction, and cell membrane stability. The Ca^2+^ content is significantly higher in the hard seed coat of wild species than that in the non-hard seed coat of cultivated soybeans; however, its exact function remains unclear [47]. Understanding the structural and compositional features of the soybean seed coat is essential for developing effective strategies to overcome seed dormancy and improve germination.

### 2.3. Molecular Characteristics of Seed Coat

Research on seed development has primarily focused on embryonic development. With an increased understanding of the importance of seed coats in seed growth and development, and the advancement of sequencing technologies, multi-omics studies application and other aspects of seed coats in different species have become increasingly prominent, enhancing our understanding of seed coat development (Table 1).

To understand the mechanism of seed development in wild type soybean and mutants with ruptured seed coats, transcriptomes of the seed coat at three stages were analyzed and differential expressions of cell wall-related proteins between the wild type, and the mutant seed coats were identified [48]. In addition to studies on the transcriptome of the *M. truncatula* seed coat before [34,49], Fu et al. explored macrosclerid cells of the *M. truncatula* seed coat at six different time points during seed development by anatomy and microarray analysis [50]. Analysis of the anatomy, metabolomics, and transcriptomics of pea seed coats revealed significant differences in surface texture, length of palisade layer cells, and seed coat thickness between wild (dormant) and cultivated (non-dormant) peas, and 14 genes possibly related to physical dormancy were predicted [5].

Gene expression in seed coats is dynamically complex. Histology, transcriptomics, and metabolomics analyses can not only identify process-related transcriptional genes and metabolite changes but also focus on biochemical pathways related to seed coats, thus delineating candidate genes that affect seed coat development. Genes encoding Type I- Inositol polyphosphate 5 phosphatase1 and E3 Ubiquitin ligase could be a preliminary association with the desirable permeability characteristics by whole genome resequencing of cultivated (soft) and wild (hard) soybeans [51]. Genome-wide association studies on seven seed dormancy traits and three bioclimatic variables identified 136 candidate genes as potential regulators of physical dormancy, most of which are involved in the biosynthesis of secondary metabolites, cell wall modification, and hormone regulation in *M. truncatula* [52]. Bulked segregant analysis (BSA) revealed that seed water uptake is associated with the candidate gene *pectin acetylesterase 8* of a single major quantitative trait locus (QTL) on Pv03 in the common bean (Phaseolus vulgaris) [53]. By analyzing the *isi2* mutant that absorbs water from the lens groove, *VsPSAT1* was identified as a candidate gene for reduced hard-seededness in *Vigna stipulacea* [54]. In hairy vetch (*Vicia villosa*), key genes with a potential role in hard-seededness, such as *KNOX4* (a class II *KNOTTED-like* homeobox *KNOXII* gene), *qHs1* (endo-1,4-β-glucanase), and *GmHs1-1* (calcineurin-like metallophosphoesterase), were further explored based on genes involved in hard-seededness from other species to query the transcriptome data [55].

**Table 1:** Sequencing studies of physical dormancy published in the past 10 years.

Species	No. of Accessions Studied	Accessions Sequenced	Tissues and Organs	Sequencing Methods	Analysis Performed	Potentially Genes	Reference(s)
*Glycine max*	2	Normal and defective seed coat	Seed coat	Illumina GaII HiSeq2000 instruments (San Diego, CA, USA)	RNA-Seq		[48]
*M. truncatula*	1	A17 ecotype	Macrosclereids cells of seed coat	Microarray	HPLC-MS		[50]
*Pisum sativum*	4	JI64, VIR320, JI92, Cameor	seed coat and pod	Illumina HiSeq2000	RNA-Seq		[5]
*Glycine max*	2	Glycine max DS9712 (soft) Glycine soja DC2008–1 (hard)	Seed	Whole genome resequencing	SNPs and InDels	Type I-Inositol polyphosphate 5 phosphatase 1, E3 ubiquitin ligase	[51]
*M. truncatula*	10/178	10/178	Seed		GWAS		[52]
*Phaseolus vulgaris*	4		Seed coat		BSA	*pectin acetylesterase 8*	[53]
*Vigna stipulacea*	384 F_2_ plants	wild-type and another accession JP252958		Whole genome resequencing	BSA	*VsPSAT1*	[54]
*Vicia villosa*	2		Seed coat		RNA-Seq	*KNOX4*, *qHs1*, *Hs1-1*	[55]

### 2.4. Mechanism of Seed Coat Development

In recent years, several genes that control physical seed dormancy have been identified in soybean, *M. truncatula,* and pea (Table 2). In soybean, a single gene, *GmHs1-1*, was found to primarily control the hard seed trait by crossing the permeable soybean variety Williams 82 with two hard-seeded varieties, PI 468916 and PI 479752, resulting in a 3:1 segregation ratio of hard to non-hard seeds [47]. *GmHs1-1* encodes a transmembrane protein, calcineurin-like metallophosphoesterase. *GmHs1-1* is primarily expressed in the Malpighian layer of the seed coat and is associated with calcium levels [47]. Another gene, *GmqHS1*, which is located adjacent to *GmHs1-1* on soybean chromosome 2, also regulates physical seed dormancy [46]. *GmqHS1* encodes an endo-1,4-β-glucanase. When *GmqHS1* was introduced to the permeable soybean variety Kariyutaka, it resulted in the accumulation of β-1,4-glucan outside the palisade layer cells, producing hard seeds [46]. This gene is likely involved in the accumulation of β-1,4-glucan derivatives, which enhances the impermeability of soybean seed coats.

In *M. truncatula*, a forward genetic approach was used to screen a large tobacco retrotransposon *Tnt1*-insertion mutant library to identify mutants lacking physical seed dormancy. The *KNOX4* gene, a class II *KNOTTED*-*like* homeobox gene, was found to control physical seed dormancy [20]. The loss of the *KNOX4* function in mutant seeds disrupts the formation of the palisade cuticle layer, allowing the seeds to easily absorb water. One of the downstream target genes of *KNOX4*, *CYP86A*, is a cytochrome P450 monooxygenase that is involved in the biosynthesis of extracellular lipid esters. The *cyp86a* mutant seeds turned blue when stained with methylene blue, indicating that the seed coat was permeable [20]. The functions of these genes have also been verified in mung bean (*Vigna radiata*) [26].

Another downstream target of *KNOX4*, *β-ketoacyl-CoA synthase 12* (*KCS12*), was significantly downregulated in *knox4* mutant seeds, and *kcs12* mutant seeds could absorb water [39]. Chemical analysis showed a significant decrease in the monomers of C24:0 lipid esters in mutant seeds, indicating that *KCS12* controls physical seed dormancy by producing ultra-long-chain lipids in the seed coat [39].

Overall, research on seed physical dormancy has mostly focused on transcriptome sequencing. Although some studies have been conducted on related genes, there is a lack of deeper systematic mechanistic investigation. The further elucidation into the inherent relationship between lipids and physical seed dormancy, the identification of genes associated with seed permeability and impermeability, and further research at the population level will help clarify the molecular mechanisms of physical seed dormancy.

### 2.5. Functions of Seed Coat

The seed coat of higher plants maintains seed integrity, protects the embryo from mechanical damage, and extends the life of seeds in a natural environment for hundreds of years [57,58,59]. The presence of a seed coat prolongs seed viability, especially for seeds harvested in field conditions [60]. In addition, the seed coat also blocks various harmful substances from the surrounding environment, improves seed resistance to fungi and bacteria, and prevents microbial invasion [61,62,63]. Furthermore, the seed coat controls gas exchange between the embryo and the environment, playing a key role in maintaining seed persistence in soil seed banks [64,65]. The seed coat also helps seeds to avoid being eaten by rodents, who prefer to detect seeds by smell [66].

Under natural conditions, many seeds germinate only under certain conditions (favorable moisture, high or low temperature, etc.) or after the seed coat has been worn off [67,68,69,70]. In agricultural production, hard seed coats result in reduced germination rates, increased seed usage, and increased agricultural costs. Therefore, breaking physical seed dormancy is crucial for agriculture production [46,47].

Seed coats develop from the maternal ovule. When non-physical-dormant materials are used as female parents and cross with hard (physical dormant) materials, the harvested seeds are non-hard seeds like the female parent and are suitable for summer sowing, whereas the seeds harvested in the next generation are hard seeds for seed overwintering. For example, hybridization experiments with *Lupinus angustifolius* showed that F_1_ seeds are non-hard seeds, while all F_2_ seeds, including those with homozygous non-hard seeds, are hard seeds.

## 3. Plants Potentially Suitable for Molecular-Level Study of Physical Seed Dormancy

Currently, the most widely studied model plants for plant genome research are *Arabidopsis thaliana* and rice (*Oryza sativa*). However, because Arabidopsis and rice seeds lack an impermeable layer, they are not good model plants for studying physical seed dormancy. In this review, we list several plant species that have been or have the potential to be used for studying physical seed dormancy. Although some physical dormancy-related genes have been identified, it is not known if they play the same roles in all species, even among leguminous species.

### 3.1. Soybean (Glycine max)

Soybean is an important legume crop. Soybean is a main source of dietary proteins and oil in animal feed and a staple food consumed by humans [71]. Wild relative *Glycine soja* varieties have a hard seed coat, whereas cultivated soybeans have a permeable seed coat, making the soybean an excellent research material for studying the role of physical dormancy during domestication. With the completion of genome sequencing *in Glycine max* and *Glycine soja*, the entire genome sequence libraries have been progressively improved, greatly promoting the research on soybean domestication and physical dormancy [72,73]. At present, both *HS1* and *qHS1* have been found to have functions in wild varieties but not in cultivated varieties, thus determining that they affect hard-seededness in soybean [46,47].

### 3.2. Medicago truncatula

Seeds of the model legume *M. truncatula* are impermeable without any scarification treatment, thus exhibiting typical physical dormancy. Using a shotgun sequencing strategy, sequences of the euchromatic regions of the eight chromosome arms of the Jemalong A17 ecotype of *M. truncatula* were determined [74,75]. Kaur et al. [76] used in situ Hi-C to reassemble and annotate the genome of another widely used ecotype, R108, of *M. truncatula*.

To accelerate genetic discovery, *M. truncatula* has been subjected to chemical, physical, and biological mutagens, including EMS, fast neutron bombardment (FNB), and transposable elements of *Nicotiana tabacum* cell type 1 (*Tnt1*) [77,78,79]. The establishment of these mutant resources has been instrumental for the discovery of the genes required for physical dormancy. Currently, two key genes (*KNOX4* and *KCS12*) that control seed physical dormancy have been identified using *M. truncatula* as a research material, fully demonstrating the advantages of *M. truncatula* in the study of physical dormancy [20,39].

### 3.3. Pea (Pisum sativum)

Pea is the second most important legume crop after soybeans and is a valuable source of dietary proteins, mineral nutrients, complex carbohydrates, and fibers, providing significant health benefits for humans [80]. Since the 18th century, pea has been used as a genetic model. In 1866, Gregor Mendel revealed the laws of inheritance through genetic analysis of different morphological pea types [81]. However, because of its large genome size (1 C~4.45 gigabases, Gb), genomic research on pea has largely lagged behind that of other legume species with smaller genomes, such as *M. truncatula* [74], *Lotus japonicus* [82], and soybean [72]. In 2019, the genome sequence of seven chromosomes of the self-fertilizing pea variety “Caméor”, characterized by its high protein content, was reported [83]. However, when comparing repetitive DNA sequences in pea, soybean, and *M. truncatula*, there is almost no sequence similarity between pea and soybean. Although the repetitive sequences between pea and *M. truncatula* are more similar, their abundance is different [84]. Wild pea species have typical physical dormancy, and potential genes related to physical seed dormancy exist in cultivated pea varieties, which will help us understand the genetic and molecular processes related to physical seed dormancy.

### 3.4. Chickpea (Cicer arietinum)

Chickpea is one of the most consumed legumes and the third largest legume crop after soybean and pea. Its high nutritional and economic values make it extremely important for food security, especially in developing countries where this crop is often grown in nutrient-poor soils under harsh climatic conditions [85]. The genome of the chickpea variety CDC Frontier was sequenced using a shotgun approach, and a genome of 300 Mbp was determined and estimated to contain 28,269 genes [86]. In addition, using next-generation sequencing platforms, artificial bacterial chromosome end sequences, and genetic maps, 520 Mbp of sequences covering 70% of the predicted 740 Mbp genome and approximately 27,571 genes were identified [87]. These data provide resources for the molecular breeding improvement of chickpeas and insight into genomic diversity and domestication.

Compared to cultivated chickpea, the seed coat of wild chickpea has significantly longer macrosclereid length and differences in lipidic substances [42]. QTL mapping analysis revealed that chickpea LG1 and LG3 control seed filling and seed coat development, thereby affecting seed shape, size, color, composition, and weight, which are key factors in determining crop yield and quality [88]. Through the long-term evolution and adaptation to extreme conditions, genes that confer tolerance to a range of abiotic stresses, including drought and cold, have been identified in chickpea [89,90]. Through an extreme domestication-related genetic bottleneck in chickpea, the genetic history of wild populations was deciphered, and the adaptive traits of the ancestral protective seed coat color were analyzed to estimate the influence of the environment on genetic structure and agronomic traits and to demonstrate the agronomic differences between wild and cultivated species [91]. Therefore, chickpea is an essential germplasm resource for the study of extreme genetic, domestication, breeding, and physical seed dormancy processes.

### 3.5. Alfalfa

As the most widely cultivated and used forage crop in the world, alfalfa has many characteristics such as tolerance to cold, drought, salt, and alkali; wide adaptability; a well-developed root system; and a strong regeneration ability. Alfalfa seeds also exhibit physical dormancy, which causes uneven germination and seedling growth [92]. To overcome this situation, some researchers use the corona discharge field to change the hydrophilicity of alfalfa seeds [93], while others use multispectral imaging and multivariate analysis to classify hard and soft seeds [92]. In practice, people coat seeds to improve yield by changing the composition of the coating agent [94]. However, these methods are not only costly and inefficient but also provide only temporary solutions. Solving the physical dormancy problem of alfalfa seeds at the molecular level is the ultimate solution.

Previously, the exploration of the genetic and genomic resources of alfalfa relied primarily on investigations into its close relative, the diploid clover, which has been sequenced. However, because they are different species with different genomes, there are clear limitations. Recently, by integrating high-fidelity single-molecule sequencing and Hi-C data, a chromosome-level alfalfa genome was generated, consisting of 32 allelic chromosomes identified using allelic gene recognition [95]. With the completion of alfalfa genome sequencing, research and molecular breeding of this important forage crop are expected to accelerate. This reference sequence will accelerate our understanding of the molecular basis of important traits, including physical dormancy, in agriculture and will support crop improvement.

### 3.6. Hairy Vetch

Kucek et al. [96] quantified the magnitude of genetic and environmental effects on physical dormancy among 1488 maternal hairy vetch plants from 18 diverse environments to explore the relationship between physical dormancy and environmental conditions during seed development. Tilhou et al. [97] reported a genome-wide association study of 1019 hairy vetch individuals to evaluate the proportion of dormant seeds. A major locus controlling seed dormancy was found (q-value: 1.29 × 10^−5^; chromosome 1: position: 63611165), which can be used by breeding programs to rapidly reduce dormancy in breeding populations [97].

## 4. Significances of Physical Dormancy

Physical dormancy of seeds is an adaptive feature for wild plant species. Physical dormancy is considered an anti-predator trait that evolved in response to powerful selection by small mammal seed predators [66]. Some studies suggest that not only predatory pressure but also several other environmental pressures were involved in increasing the fitness of species producing seeds with PY [98].

Field experiments have shown that physical dormancy reduces seed germination rates and leads to a significant decrease in wild pea yield [99]. In the processing of plant oils and soy products, physical dormancy affects seed oil yield and soy product quality [100,101]. In most food legumes, seeds without physical dormancy absorb moisture more easily, making them easier to cook [100,101].

Loss of physical seed dormancy is crucial for the domestication of many leguminous crops and is one of the key traits for legume crop domestication [102,103]. In general, wild leguminous plant seeds exhibit physical dormancy, whereas modern cultivated varieties often lose physical dormancy [24,25,104,105]. In wild fruits, the loss of physical dormancy is a basic characteristic that ensures seed dispersal. In cultivated fruits, people often choose varieties with physical dormancy to reduce seed coat (fruit skin) breakage and facilitate fruit harvest and storage [106,107]. Research on physical dormancy and other domestication-related changes in seeds will helps to enhance understanding of evolution and domestication [108,109].

## 5. Conclusions and Perspectives

In this review, we discussed the developmental mechanisms of seed coats and their composition, which influence physical dormancy, and suitable research materials for studying this physiological phenomenon. Although physical dormancy has long been recognized, it was not until 2015 that the first gene responsible for hard seededness was discovered in soybean. Due to the lack of suitable research materials, there is much less research on physical dormancy than on physiological dormancy in Arabidopsis and rice. With more legume resources becoming available in recent years, some exciting studies on physical dormancy have been reported, although many more remain to be investigated. Further gene identification in this field in the future will provide more insight into the real mechanisms of physical dormancy and provide more potential for legume crop improvement. In the past, since the mechanism of physical dormancy is unclear, research on the relationship of physical dormancy and plant pathogens is scarce. With more breakthroughs in physical seed dormancy, the relationship between physical seed dormancy and plant pathogens is another field to pay attention to. The use of genome editing technology is a promising approach to improve the hard and fruity traits of leguminous forage seeds. With the identification and characterization of more and more genes involved in physical dormancy in model legumes, it is feasible to edit these genes in leguminous and other crops with physical dormancy. The advantage of genome editing technology is that it can effectively replace the long hybridization and screening process required for traditional domestication, thereby shortening the domestication process.

## Figures and Tables

**Table 2:** Genes that regulate physical dormancy.

**Gene**	**Orthologs in Arabidopsis**	**Species**	**Gene Loci**	**Gene Function**	**Causative Change**	**Gene Identification Methods**	**Reference(s)**
*GmHs1-1*		*Glycine max*	*Glyma.02g269500*	Metallophospholipase	Accumulation Ca^2+^	Mapping	[47]
*qHS1*			*Glyma.02g43680* (*Glyma.02g269400*)	Endo-1,4-β-glucanase	Accumulation β-1,4-Glucan	Mapping	[46]
*KNOX4*	*KNAT7*	*M. truncatula* *Vigna radiata*	*Medtr5g011070*	A class II KNOX gene	Change palisade cuticle layer	TAIL-PCR	[20,56]
*KCS12*	*KCS12*	*M. truncatula*	*Medtr2g096210*	β-Ketoyl CoA Synthase	Control the production of VLCFAs in seed coat	Microarray	[39]
